# Arthroscopic Quadriceps Tendon Repair: Two Case Reports

**DOI:** 10.1155/2015/937581

**Published:** 2015-02-28

**Authors:** Hidetomo Saito, Yoichi Shimada, Toshiaki Yamamura, Shin Yamada, Takahiro Sato, Koji Nozaka, Hiroaki Kijima, Kimio Saito

**Affiliations:** ^1^Department of Orthopedic Surgery, Akita University Graduate School of Medicine, Akita 010-8543, Japan; ^2^Sapporo Sports Clinic, Sapporo 060-0001, Japan

## Abstract

Recently, although some studies of open repair of the tendon of the quadriceps femoris have been published, there have been no reports in the literature on primary arthroscopic repair. In our present study, we present two cases of quadriceps tendon injury arthroscopically repaired with excellent results. Case 1 involved a 68-year-old man who was injured while shifting his weight to prevent a fall. MRI showed complete rupture at the insertion of the patella of the quadriceps tendon. The rupture was arthroscopically repaired using both suture anchor and pull-out suture fixation methods via bone tunnels (hereafter, pull-out fixation). Two years after surgery, retearing was not observed on MRI and both Japan Orthopedic Association (JOA) Knee and Lysholm scores had recovered to 100. Case 2 involved a 50-year-old man who was also injured when shifting his weight to prevent a fall. MRI showed incomplete superficial rupture at the insertion of the patella of the quadriceps tendon. The rupture was arthroscopically repaired using pull-out fixation of six strand sutures. One year after surgery, MRI revealed a healed tendon and his JOA and Lysholm scores were 95 and 100, respectively. Thus, arthroscopic repair may be a useful surgical method for repairing quadriceps tendon injury.

## 1. Introduction

Quadriceps femoris tendon rupture represents a well-documented injury to the extensor mechanism of knee joint, affecting predominantly men over 40 years of age in those with degenerative changes or systemic disease [1–4]. Systemic diseases such as lupus erythematosus, diabetes, gout, hyperparathyroidism, uremia, and obesity have been associated with disruption of the quadriceps mechanism [5].

Over the years, the repair techniques have progressed from simple suture with catgut or silk to wire-reinforced repair, pull-out suture fixation through patella, suture anchor fixation, tendon lengthening repair, Scuderi technique, allograft, autograft, and synthetic materials [1, 4, 6–12]. However, no literature has reported the result using arthroscopy.

The purpose of reporting spontaneous quadriceps tendon rupture cases was to describe a new surgical procedure using arthroscopy which indicated the positive effect of stable fixation followed by early range of motion exercise on the result of treatment.

## 2. Case Reports

### 2.1. Case  1

**Figure 1:**
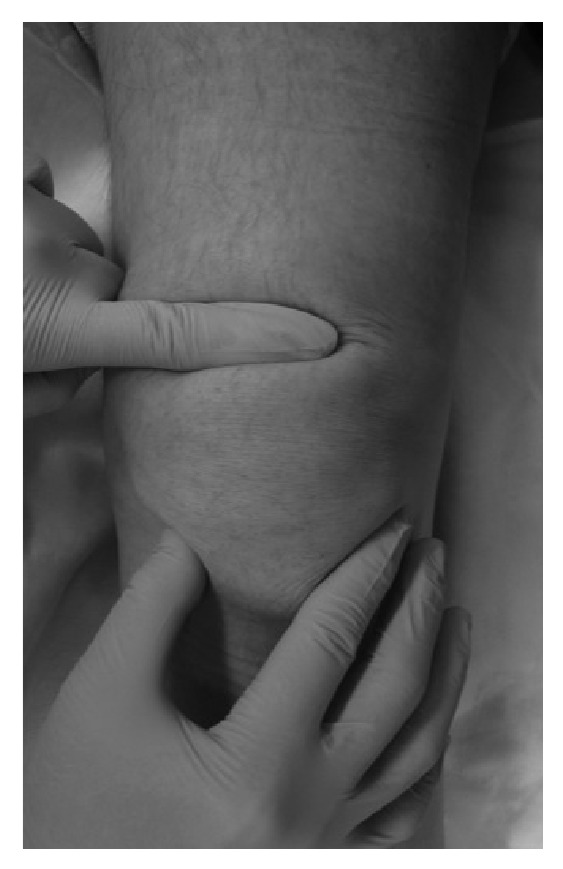
Macroscopic photograph of clinical findings. A tendon defect was (1.5 cm in size) proximal to the patella that was palpable.

**Figure 2:**
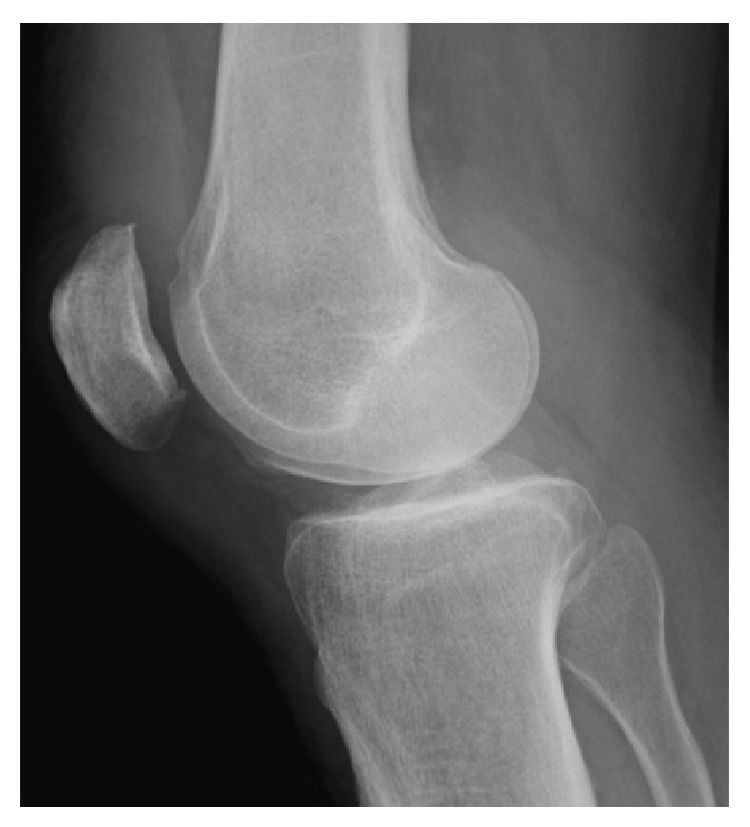
Lateral plain radiograph. Osteophyte formation was observed in the upper pole of the patella.

**Figure 3:**
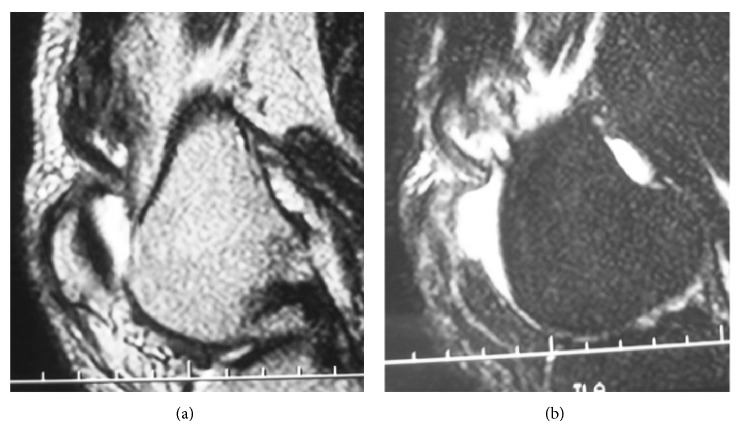
MRI findings. (a) A proton density-weighted image. Continuity of the quadriceps tendon to the patella was completely disrupted. (b) A T2-weighed fat-suppressed image. There was a high-intensity area in the midsubstance of the quadriceps tendon.

The subject was a 68-year-old male artist with a body mass index (BMI) of 29.1. He had no relevant medical history. When he was walking on an icy street and shifted his weight to prevent a fall, he felt severe pain in the left knee and had difficulty in walking. He visited our facility with a chief complaint of left knee pain. At the initial examination, a tendon defect at proximal tip to the patella was palpable (Figure 1). Active straight leg raising was not possible and the extension lag sign was positive. A lateral plain radiograph image showed osteophyte formation in the upper pole of the patella (Figure 2). Magnetic resonance imaging (MRI) using proton density-weighted images showed interrupted continuity of the quadriceps tendon and the patella (Figure 3(a)). A fat-suppressed T2-weighted image revealed signal changes in the quadriceps tendon (Figure 3(b)). Based on these findings, the patient was diagnosed with a complete rupture of the quadriceps tendon for which he underwent arthroscopic repair.

**Figure 4:**
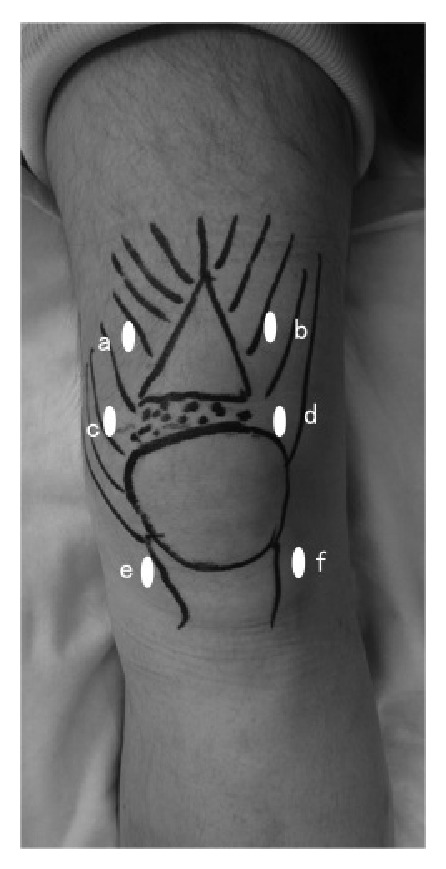
Location of arthroscopic portals. Portal locations in the left knee. (a) Medial far proximal portal. (b) Lateral far proximal portal. (c) Medial suprapatellar portal. (d) Lateral suprapatellar portal. (e) Medial parapatellar portal. (f) Lateral parapatellar portal.

During the surgery, a perfusion pump (Smith & Nephew KK, Tokyo, Japan) was used but no air tourniquet. Medial and lateral parapatellar portals, medial and lateral suprapatellar portals (patellar upper pole level), and medial and lateral far proximal portals were prepared (Figure 4). The parapatellar and the suprapatellar portals were used as viewing and working portals, and the far proximal portal was used as a working portal. After the site of the rupture was identified, the medial site was sutured with suture anchor. The anchor was inserted via medial far proximal portal. Two bone tunnels at the central and lateral upper rim of the patella were created via stub incisions and a pull-out suture fixation via the bone tunnels (hereafter, pull-out fixation) was applied for repair using two high-strength threads for each bone tunnel (four threads in total via pull-out fixation). A suture thread was passed through the quadriceps tendon using a suture grasper (Ideal Suture Grasper, 60°; DePuy Synthes Mitek Sports Medicine, Raynham, MA).

**Figure 5:**
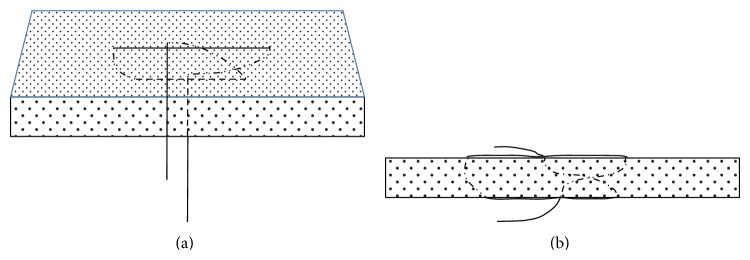
Schema of modified Mason–Allen method.

**Figure 6:**
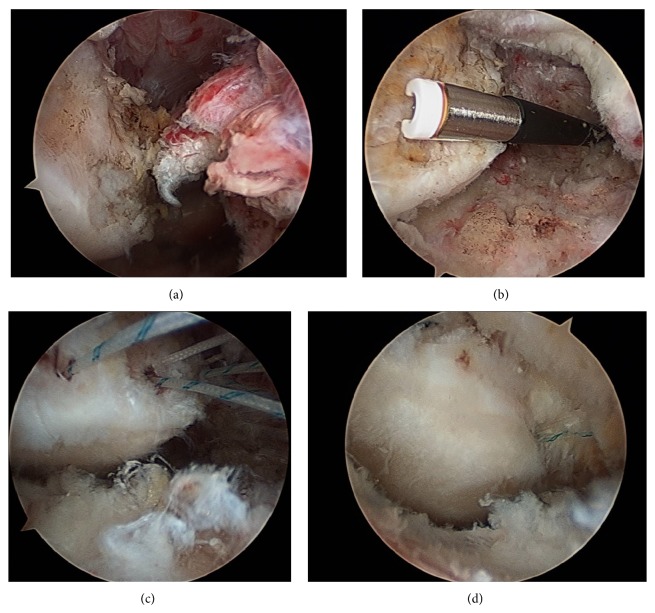
Intraoperative arthroscopic findings. (a) Arthroscopic images from the lateral suprapatellar portal. Left, patella; right, ruptured tendon. (b) Arthroscopic view from the lateral suprapatellar portal. A high-frequency radiofrequency device was inserted via the medial suprapatellar portal to debride synovia and ruptured fibers. (c) Arthroscopic view from the lateral suprapatellar portal. A suture anchor was inserted at the medial upper rim of the patella. Two bone tunnels were created at the central and lateral upper rim of the patella and two strands of #2 Ultrabraid sutures were inserted into each bone tunnel. (d) Arthroscopic view from the lateral suprapatellar portal after the repair. Continuity of the patella and the quadriceps tendon was obtained.

**Figure 7:**
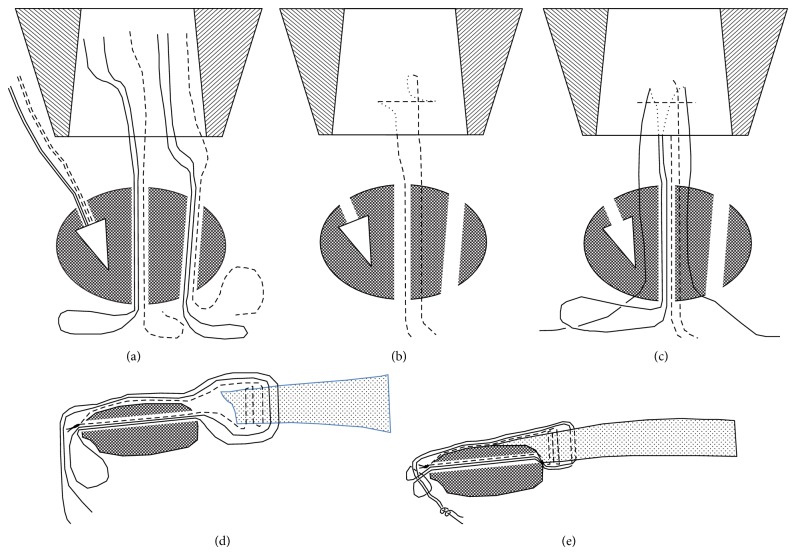
Schema for the arthroscopic suture in Case  1. (a) Two threads (a dotted line for the Mason–Allen method and a solid line for the double-loop sliding knot) were used for the central and lateral bone tunnels. (b) The thread (dotted line) in the central bone tunnel holding the tendon by the Mason–Allen method was passed through the anterior region and inside of the tunnel of the patella and was pulled out to the patellar lower rim. (c) The thread (solid line) holding the tendon over and proximal to the roof of the Mason–Allen method was next passed through the anterior region and inside of the tunnel of the patella and was pulled out to the patellar lower rim. The procedures used in (b) and (c) were also performed in the lateral bone tunnel. (d) and (e) Each thread gathered at the patellar lower rim was tied.

To create the bone tunnels for the pull-out fixation, a “2.4 mm × 15” Graduated Drill-Tip Passing Pin (Smith & Nephew Inc, Andover, MA) was used. Two high-strength threads (Ultrabraid; Smith & Nephew Inc., Andover, MA) were passed through each bone tunnel of the patella with the eyelet of the 2.4 mm drill pin; one of them was looped before passing. Subsequently, one end of the high-strength thread was passed through the quadriceps tendon using the modified Mason–Allen suture method (Figure 5), was slid over the patellar surface using suture retriever (Smith & Nephew Inc., Andover, MA), and was tied at the patellar lower pole via a small incision with the other side of the thread pulled out from the bone tunnel. This procedure was performed for two high-strength threads. The residual two looped high-strength threads were tightened using a double-loop sliding knot. The fixity was favorable and the continuity of the tendon and patella was obtained (Figures 6(a)–6(d)). The pull-out fixation schema is depicted in Figures 7(a)–7(e). Operation time was 3 hours and 25 minutes.

Postoperatively, a knee brace was applied with the leg extended. Continuous passive motion (CPM) was started 1 week after surgery, one-third partial weight bearing 6 weeks after surgery, and full-weight bearing 8 weeks after surgery. ROM exercises were carefully conducted respecting the repair site to be able to flex 90 degrees at 8 weeks after surgery and 120 degrees at 12 weeks and to perform full flexion at 6 months.

**Figure 8:**
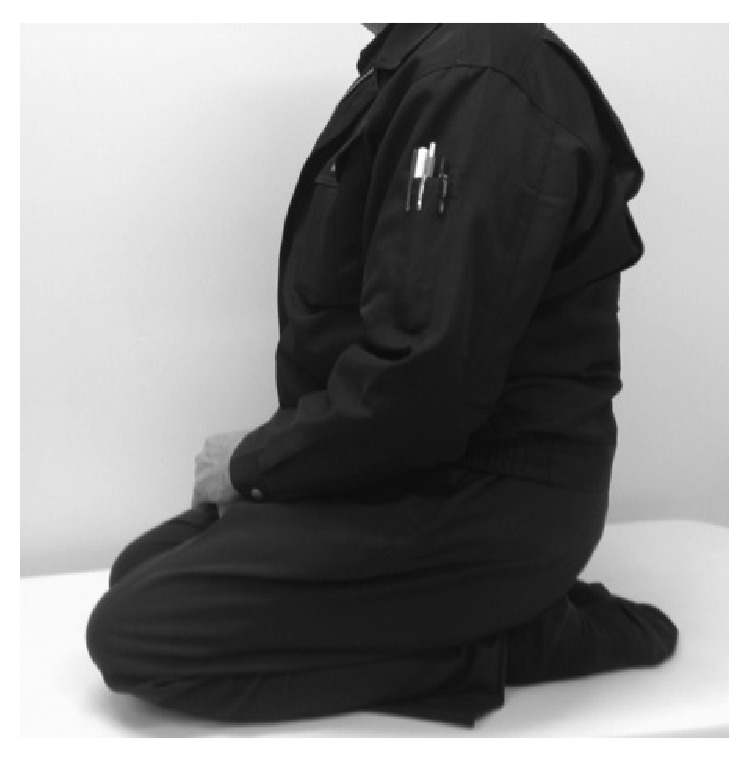
The patient could sit on his heels 2 years after surgery.

**Figure 9:**
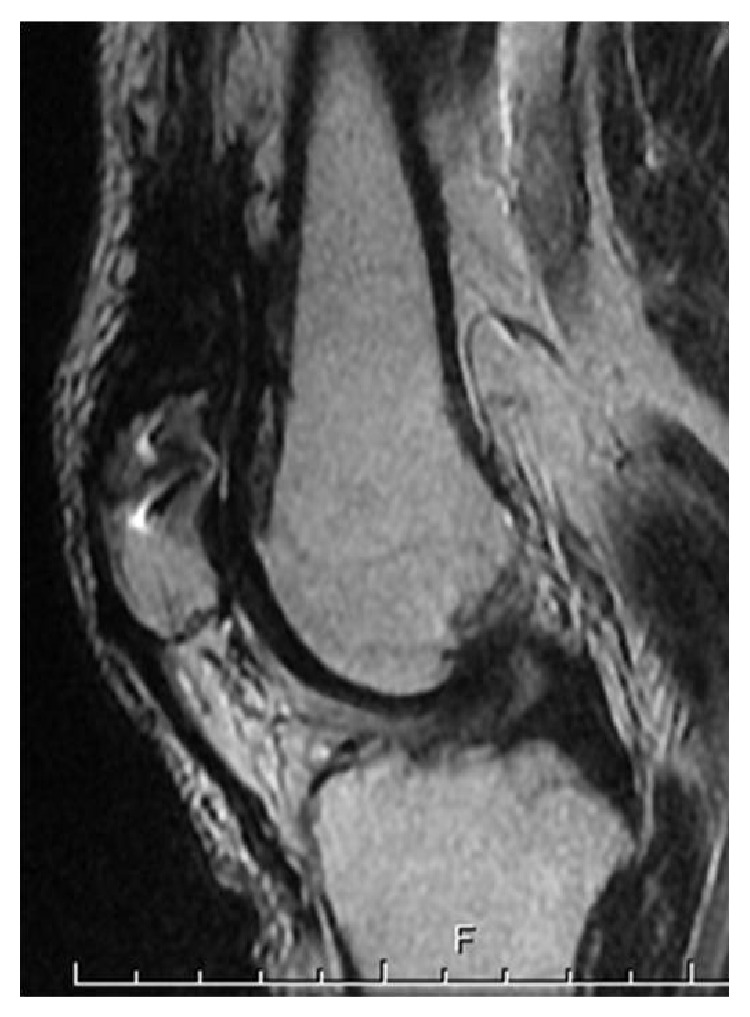
Sagittal postoperative MRI proton density-weighted image. Continuity of the tendon was maintained.

The patient could sit on his heels 6 months after surgery (Figure 8). Two years after surgery, both his Lysholm and Japan Orthopedic Association (JOA) scores were 100 and favorable continuity of the quadriceps tendon and patella was seen on MRI (Figure 9). Artistic activities in a deep flexed knee position (such as a Japanese tea ceremony) could be performed without problems and the patient could walk 20 km a day. The patient was satisfied with these results.

### 2.2. Case  2

**Figure 10:**
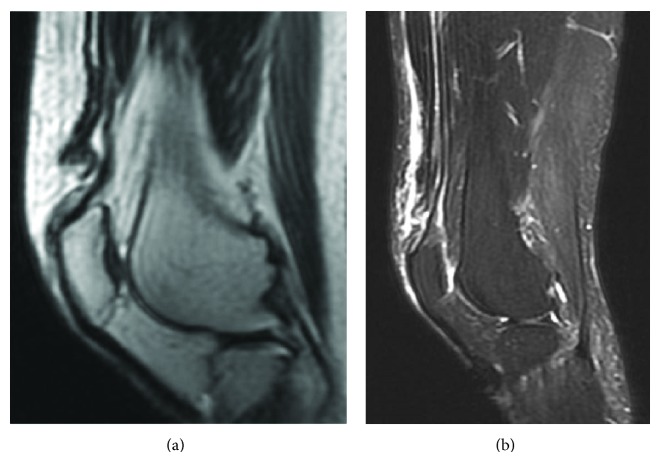
Preoperative MRI image in Case  2. (a) A sagittal proton density-weighted image showing maintained continuity of the deep layer of the quadriceps tendon and patella, in addition to disruption and deflection of the superficial layer. (b) Short tau inversion recovery (STIR) imaging showing the same changes as in (a).

This subject was a 50-year-old male sales representative with a BMI of 34.2. He had no relevant medical history. When he was walking and shifted his weight to prevent a fall, he felt pain in the left knee and had difficulty walking. He visited our facility with a chief complaint of left knee pain. At the initial examination, tenderness and a tendon defect at proximal to the patellar upper pole of the left knee were observed. Active straight leg raising was impossible and the extension lag sign was positive. MRI proton density-weighted images with and without fat suppression showed disruption of the superficial layer of the quadriceps tendon and maintained continuity of the deep layer (Figures 10(a) and 10(b)). Based on these findings, he was diagnosed with a partial rupture of the superficial layer of the quadriceps tendon for which he underwent arthroscopic repair.

**Figure 11:**
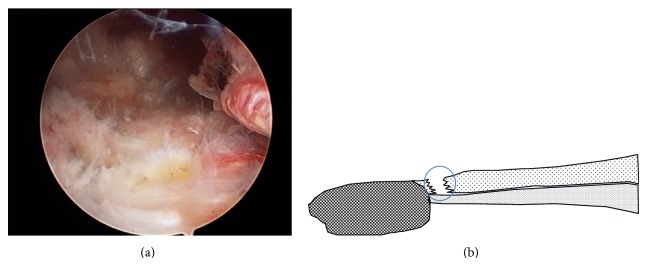
Arthroscopic view and schema from the lateral suprapatellar portal in the left knee. (a) The free space between the superior rim of the patella (left) and the torn tendon (right) was caused by the disruption of the superficial layer by the quadriceps tendon rupture. The intact deep layer is visible on the lower side. (b) The schema of (a).

Medial and lateral parapatellar portals, medial and lateral suprapatellar portals, and medial and lateral far proximal portals were prepared for the surgery. The parapatellar and the suprapatellar portals were used as arthroscopic and work portals. The far proximal portal was used as a work portal. The ruptured region could not be arthroscopically observed from the suprapatellar pouch but could be identified between the deep layers of the intact quadriceps tendon and inferior to the superficial fascia of the quadriceps. We identified the torn tendon as the superficial layer of the quadriceps tendon (Figures 11(a) and 11(b)). Three bone tunnels were made at internal, medial, and external sites via stub incisions and pull-out fixation was applied for restoration using two high-strength threads for each bone tunnel (six threads in total).

**Figure 12:**
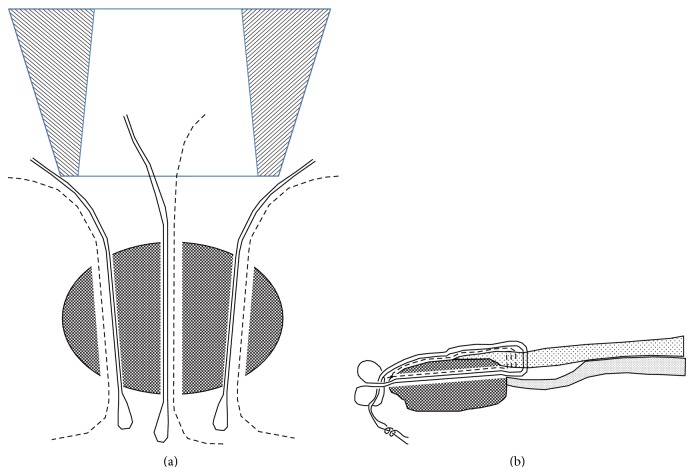
Schema of the arthroscopic suture in Case  2. (a) Two threads (a dotted line for the Mason–Allen method and a solid line for the double-loop sliding knot) were used for the internal, medial, and external bone tunnels. (b) As with Case  1, after the threads were passed through both the superficial layer and the inside of the tunnel of the patella, they were then tied at the lower rim. Subsequently, only the superficial layer was repaired.

To create the bone tunnels for the pull-out fixation, a “2.4 mm × 15” Graduated Drill-Tip Passing Pin (Smith & Nephew Inc, Andover, MA) was used. Two high-strength threads (Ultrabraid; Smith & Nephew Inc., Andover, MA) were passed through each bone tunnel with the eyelet of the 2.4 mm drill pin; one of them was looped before passing. One end of the high-strength thread was passed through the quadriceps tendon with the modified Mason–Allen suture method, was slid over the patellar surface using suture retriever (Smith & Nephew Inc., Andover, MA), and then was tied at the patellar lower pole with the other side of the thread pulled out from the bone tunnel via a small incision. This procedure was performed for three high-strength threads. The three residual looped high-strength threads were tightened using a double-loop sliding knot. The fixity was favorable and the continuity of the tendon and patella was observed under arthroscopic vision. The schema of pull-out fixation is presented in Figures 12(a) and 12(b). Operation time was 2 hours and 51 minutes.

**Figure 13:**
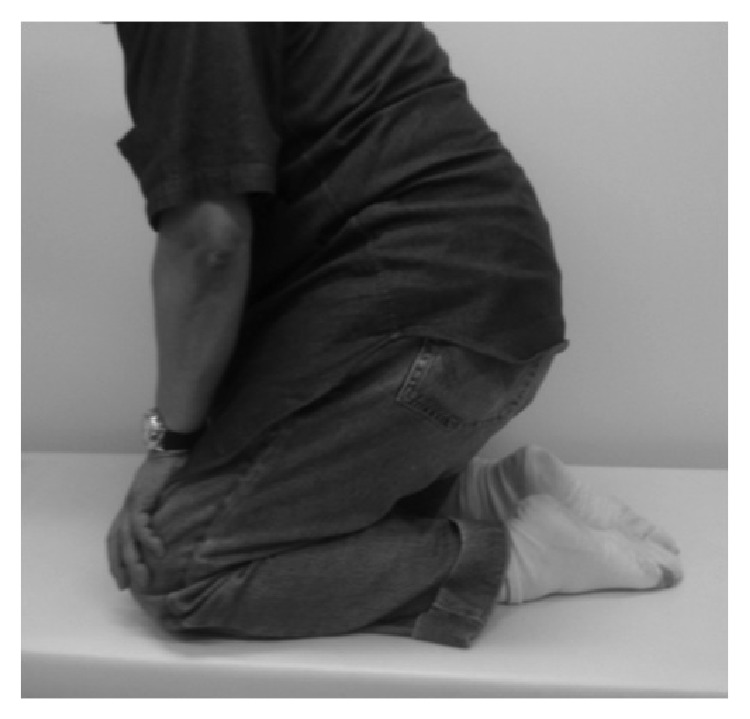
Flexion was still insufficient and rehabilitation is ongoing one year after the surgery.

**Figure 14:**
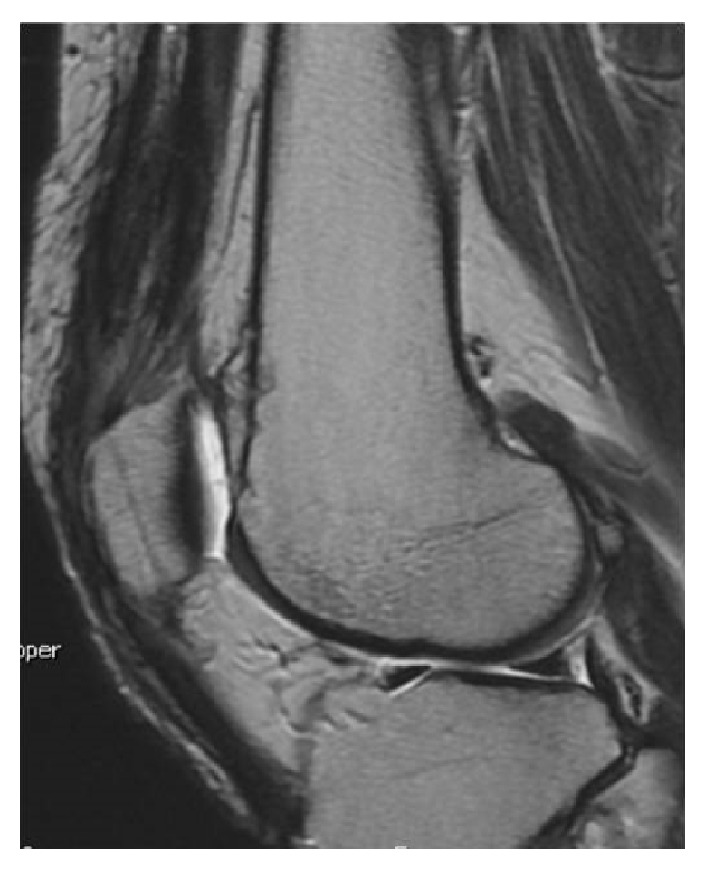
The architecture of the repaired quadriceps tendon seemed almost normal on a sagittal postoperative MRI proton density-weighted image.

Postoperatively, knee brace fixation was applied with the leg extended. CPM was started 1 week after surgery, one-third partial weight bearing 6 weeks after surgery, and full-weight bearing 8 weeks after surgery. One year after surgery, the Lysholm and JOA scores were 100 and 95, respectively, and mild limitation of flexion range was observed (Figure 13). MRI showed favorable continuity of the quadriceps tendon and patella (Figure 14). The patient had no giving way incidents and could perform daily activities and work without difficulty.

## 3. Discussion

To the best of our knowledge, there have been no previous reports on the arthroscopic repair of a quadriceps tendon rupture. Quadriceps tendon rupture, a disruption of the knee extension mechanism, is a severe injury resulting in disabilities and dysfunction that should be immediately corrected using open repair [1, 8, 11, 13]. Pull-out fixation of the patella has conventionally been performed for quadriceps tendon rupture, and good to excellent results are reported [1, 8, 11, 13]. In addition, a repair method using suture anchors has been reported [11, 14–16]. Bushnell et al. [14] reported good to excellent outcomes with suture anchors applied to the patellar upper pole to repair the quadriceps tendon in five knees. The advantage of suture anchors is due to the so-called “dead length” concept, in which the thread sutured at the repaired tendon site becomes shorter than the thread passing through the patellar bone tunnel and a gap in the thread tensile caused by repeated tensile loading does not easily occur [17]. Furthermore, surgery using anchors is less invasive since the skin incision is smaller [14].

On the other hand, Hart et al. [15] biomechanically compared the ultimate tensile loads under cyclic load between suture anchor fixation using four threads and pull-out fixation using two threads. The ultimate tensile load was 447 N in the suture anchor group, which was less than the 591 N in the pull-out fixation group. All constructs failed in the suture anchor group, with breakage of the eyelet. In contrast, there was a breakage of the suture itself in the pull-out fixation group, suggesting that the pull-out fixation was significantly biomechanically stronger than the suture anchor fixation. In our current report, Case  1 underwent both suture anchor and pull-out fixations under arthroscopic vision. Both fixation methods were technically viable under arthroscopic vision, and tendon repair was performed mainly with the pull-out fixation, which was less invasive than the conventional tendon repair under direct vision. The patient's satisfaction level was also high. In Case  2, because the pull-out fixation was biomechanically superior to the suture anchor fixation, we performed only the pull-out fixation using six high-strength threads under arthroscopic vision. Hart et al. [15] sutured the quadriceps tendon using two strands of #2 fiber wires (Arthrex, Inc., Naples, FL) via the Krakow method, whereas we used six strands of #2 Ultrabraid (Smith & Nephew Endoscopy, Inc., Tokyo, Japan). Hence, the initial strength in our cases was thought to be higher than in their cases.

As tendon suture methods, the so-called “pull-and-hold” or “tendon-holding” approaches, such as the Kessler or Krakow methods, in which the threads do not cut the tendon, have been developed. The modified Mason–Allen method is one such method. It is reported to be useful for maintaining the tendon within the tendon suture [18]. This method was mainly developed in the field of arthroscopic shoulder joint surgery and there is insufficient biomechanical evidence for its use in loading joints such as the knee joint. However, it has superior evidence for tendon maintenance compared with simple sutures. In our cases, we applied the modified Mason–Allen and the double-loop sliding knot methods so as not to cut the tendon and in order to improve the initial strength.

Many investigators have reported that the shorter the period between injury and surgery, the better the clinical outcome, regardless of surgery method, age, or BMI, in patients with quadriceps tendon with no underlying diseases [3, 19]. Delayed diagnosis leads to unsatisfactory clinical outcomes [19]. Konrath et al. [1] suggested that, in cases where surgical treatment was delayed for 2 or more weeks, the percentage of patients achieving complete functional recovery decreased from 50% to 21.4%. Therefore, early diagnosis and early restoration are important for preventing disability.

Regarding postoperative rehabilitation, it has been reported that prolonged knee immobilization for 6 to 8 weeks after primary suture was necessary to allow complete healing of the repair and to ensure an acceptable outcome [3]. Konrath et al. [1] reported that self-operating flexion exercise in a prone position should be started from the early phase after primary suturing with pull-out fixation to facilitate tendon fusion. Moreover, early active exercise becomes possible by augmenting the tendon with soft wires, artificial ligament, or nonabsorbable sutures [19–21]. On the other hand, some biomechanical studies suggested that, because the initial strength is insufficient after primary suture of the patella using pull-out and suture anchor fixation methods, the tendon should be fixed at the completely extended position and exercises, except for isometric exercise, should be started after tendon fusion to prevent the development of anastomotic leakage at the sutured site [15, 22]. In addition, postoperative rehabilitation should be determined according to the presence of the underlying diseases, influence of body weight, period between injury and surgery, and surgical methods. In our cases, because the BMIs of the patients were high, we postoperatively immobilized the knees in an extended position to avoid retearing due to early weight bearing. CPM was started 1 week after surgery, one-third partial weight bearing 6 weeks after surgery, and full-weight bearing 8 weeks after surgery. ROM exercises were carefully conducted with respect to the repair site to be able to flex 90 degrees at 8 weeks after surgery, 120 degrees at 12 weeks and full flexion at 6 months. Two years after surgery, Case  1 could sit on his heels. Although Case  2 could not sit on his heels, his knee joint function had recovered to the same level as that before injury (Figure 14).

### 3.1. Advantages and Disadvantages, Difficulties

Advantages of arthroscopic surgery have been reported such as minimizing soft tissue trauma during surgeries that caused postoperative impairment, providing better vision [23, 24]. Consequently, alleviating postoperative pain, early functional recovery, and shorter hospital stay were expected that had a positive effect on medical economy. On the other hand, arthroscopic surgery has been technically demanded requiring significant arthroscopic skills, fairly longer learning curve, and longer operative time, even if surgeons were well trained, and many instruments or specially designed devises have been developed. However, in this present report, we could conduct similar postoperative rehabilitation after arthroscopic repair compared with open surgeries that had been already reported. Thus, we believe that such sorts of arthroscopic surgery would be one of methods to treat patients with quadriceps tendon injury.

## 4. Conclusions

Arthroscopic repair of quadriceps tendon rupture can provide excellent results.

## Supplementary Material

Arthroscopic quadriceps tendon repair of case 2.
